# [2-(3,4-Di­meth­oxy­phen­yl)eth­yl](3-{*N*-[2-(3,4-di­meth­oxy­phen­yl)eth­yl]carbamo­yl}prop­yl)aza­nium chloride dihydrate

**DOI:** 10.1107/S1600536814001998

**Published:** 2014-01-31

**Authors:** Abdusalom Sh. Saidov, Kambarali K. Turgunov

**Affiliations:** a S. Yunusov Institute of the Chemistry of Plant Substances, Academy of Sciences of Uzbekistan, Mirzo Ulugbek Str. 77, Tashkent 100170, Uzbekistan

## Abstract

The asymmetric unit of the title hydrated salt, C_24_H_35_N_2_O_5_^+^·Cl^−^·2H_2_O, contains one organic cation that has its protonation site at the amine function, one chloride anion and two lattice water mol­ecules. In the crystal, one pair of lattice water mol­ecules and two chloride anions form a four-membered centrosymmetric hydrogen-bond cycle. In addition, O—H⋯O, N—H⋯O and N—H⋯Cl hydrogen bonds involving the N—H groups, the water mol­ecules and the C=O group are observed. As a result, a hydrogen-bonded layer parallel to (100) is formed. The thickness of such a layer corresponds to the length of the *a* axis [21.977 (3) Å].

## Related literature   

For standard bond lengths, see: Allen *et al.* (1987 ▶). For the synthesis of related compounds, see: Bentley (2006 ▶); Saidov *et al.* (2013 ▶). For the crystal structure of a related compound, see: Peters *et al.* (1994 ▶).
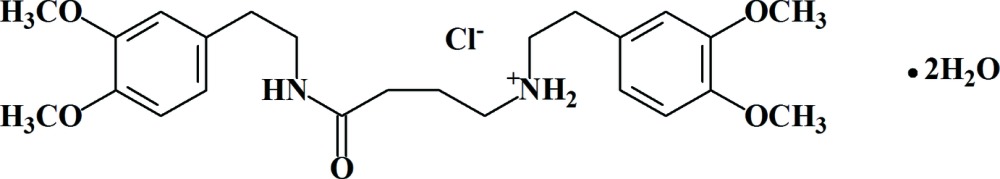


## Experimental   

### 

#### Crystal data  

C_24_H_35_N_2_O_5_^+^·Cl^−^·2H_2_O
*M* *_r_* = 503.02
Monoclinic, 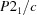

*a* = 21.977 (3) Å
*b* = 12.2295 (10) Å
*c* = 10.2217 (9) Å
β = 93.490 (9)°
*V* = 2742.2 (5) Å^3^
*Z* = 4
Cu *K*α radiation
μ = 1.59 mm^−1^
*T* = 295 K
0.60 × 0.40 × 0.35 mm

#### Data collection  

Oxford Diffraction Xcalibur Ruby diffractometer
Absorption correction: multi-scan (*CrysAlis PRO*; Oxford Diffraction, 2009 ▶) *T*_min_ = 0.599, *T*_max_ = 1.000
11225 measured reflections
4860 independent reflections
3012 reflections with *I* > 2σ(*I*)
*R*_int_ = 0.035

#### Refinement  

*R*[*F* ^2^ > 2σ(*F* ^2^)] = 0.046
*wR*(*F* ^2^) = 0.131
*S* = 0.97
4860 reflections
339 parameters
H atoms treated by a mixture of independent and constrained refinement
Δρ_max_ = 0.20 e Å^−3^
Δρ_min_ = −0.24 e Å^−3^

### 

Data collection: *CrysAlis PRO* (Oxford Diffraction, 2009 ▶); cell refinement: *CrysAlis PRO*; data reduction: *CrysAlis PRO*; program(s) used to solve structure: *SHELXS97* (Sheldrick, 2008 ▶); program(s) used to refine structure: *SHELXL97* (Sheldrick, 2008 ▶); molecular graphics: *XP* in *SHELXTL* (Sheldrick, 2008 ▶); software used to prepare material for publication: *publCIF* (Westrip, 2010 ▶).

## Supplementary Material

Crystal structure: contains datablock(s) I, GLOBAL. DOI: 10.1107/S1600536814001998/wm2799sup1.cif

Structure factors: contains datablock(s) I. DOI: 10.1107/S1600536814001998/wm2799Isup2.hkl

Supporting information file. DOI: 10.1107/S1600536814001998/wm2799Isup3.cml

CCDC reference:

Additional supporting information:  crystallographic information; 3D view; checkCIF report

## Figures and Tables

**Table 1:** Hydrogen-bond geometry (Å, °)

*D*—H⋯*A*	*D*—H	H⋯*A*	*D*⋯*A*	*D*—H⋯*A*
N1—H1⋯Cl1^i^	0.87 (2)	2.44 (2)	3.300 (2)	173 (2)
O2*W*—H1*W*2⋯O3	0.95 (5)	1.75 (5)	2.704 (3)	179 (6)
N2—H2⋯Cl1	0.92 (3)	2.22 (3)	3.127 (2)	169 (3)
O2*W*—H2*W*2⋯O1*W* ^ii^	0.80 (4)	1.97 (4)	2.767 (4)	171 (4)
N2—H3⋯O2*W* ^iii^	0.99 (2)	1.72 (2)	2.709 (3)	177 (2)
O1*W*—H1*W*1⋯Cl1^i^	0.87 (5)	2.31 (5)	3.177 (3)	177 (6)
O1*W*—H2*W*1⋯Cl1	0.91 (4)	2.31 (4)	3.194 (4)	164 (3)

Symmetry codes: (i) 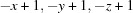; (ii) 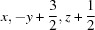; (iii) 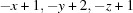.

**Fig. 1.**
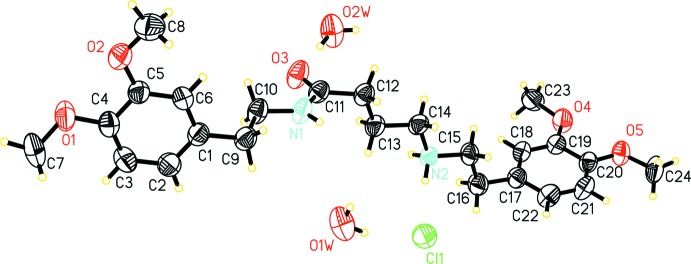
The molecular entities of the title compound with displacement ellipsoids drawn at the 50% probability level.

**Fig. 2.**
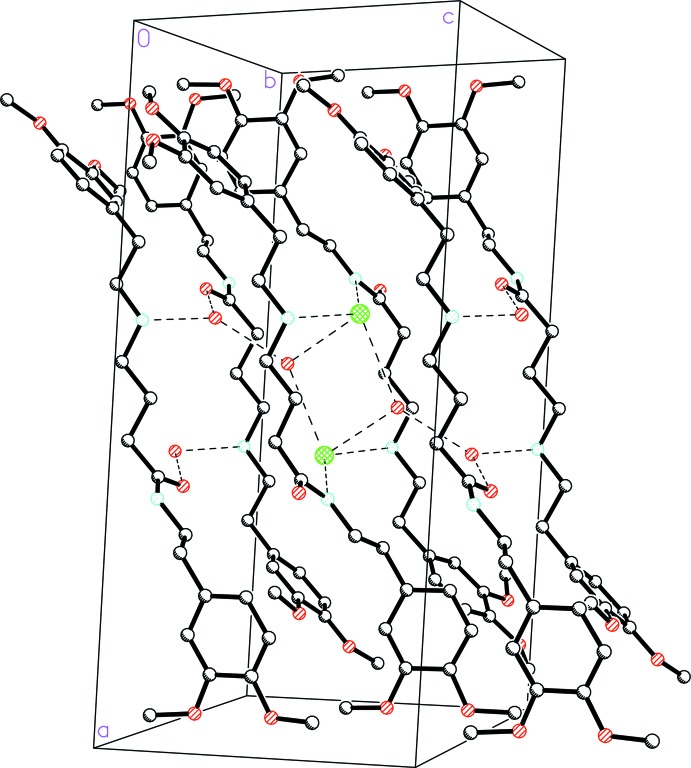
The crystal packing of the title compound showing hydrogen bonds (dashed lines)

## supplementary crystallographic information

### 1. Comment

The title compound, C_24_H_35_N_2_O_5_^+^^.^Cl^-^(H_2_O)_2_, was isolated as an intermediate product in the synthesis of isoquinoline alkaloid analogues (Saidov *et al.*, 2013). Similar compounds have been synthesized (Bentley, 2006), or their structures characterized (Peters *et al.*, 1994).

A perspective view of the moleculular entities of the title compound, showing the atomic numbering scheme, is given in Fig. 1. Bond lengths and angles are in normal ranges (Allen *et al.*, 1987). The organic molecule contains two N atoms, amidic and aminic. The N atom on an amide is usually less nucleophilic than the N atom of an amine, due to the resonance stabilization of the N atom lone-pair provided by the amide carbonyl group. Therefore, in the cation the protonization of the amino N atom is observed.

In the crystal structure, two chloride anion and one lattice water molecule form a centrosymmetric four-membered hydrogen-bonding cycle (Fig. 2). The protonated organic molecules are bridged by N—H···Cl and C═O···H—O(w) hydrogen bonds (Table 1). As a result, hydrogen-bonded layers parallel to (100) are formed that have a thickness corresponding to the length of the *a* axis.

### 2. Experimental

To a solution of 2.0 g. (0.004 mol) *N*,*N*-(3,4-dimethoxyphenyl ethyl)succindiamide in 30 ml absolute benzene was added 6.7 g (0.04 mol) POCl_3_. The reaction mixture was boiled for 2 h. Benzene and excess POCl_3_ were then removed under reduced pressure, and the residue was dissolved in 30 ml methanol. To the received solution was added 3.8 g (0.1 mol) NaBH_4_ at 273-278 K under ice cooling. Then methanol was removed, the residue dissolved in water and extracted with chloroform. From the chloroformic layer were obtained three componds with *R*_f_ 0.9, 0.5 and 0.2 (title compound) (chloroform:methanol=8:1). The compounds were isolated by column chromatography (silica gel); 0.075 g of the title compound were obtained with a m.p. 406-408 K. IR (KBr, ν, cm^-1^): 3434, 3258, 2940, 1651, 1590, 1519. Crystals suitable for X-ray diffraction analysis were obtained from a chloroform–methanol (8:1) mixture by slow evaporation.

### 3. Refinement

Carbon-bound H atoms were placed geometrically and treated as riding on their parent atoms, with C—H distances of 0.93 Å (aromatic), 0.97 Å (methylen), 0.96 Å (methyl) and were refined with *U*_iso_(H)=1.2Ueq(C) for aromatic and methylen H atoms, *U*_iso_(H)=1.5Ueq(C) for methyl H atoms. N-bound H atoms and water H atoms involved in the intermolecular hydrogen bonding were found by difference Fourier synthesis and refined isotropically [N1–H1=0.86 (2) Å, N2–H2 0.92 (3) Å, N2–H3 0.99 (3) Å, O1W–H1W1= 0.86 (5) Å, O1W–H2W1= 0.91 (5) Å, O2W–H1W2= 0.96 (4) Å, O2W–H2W2= 0.80 (4) Å].

### Crystal data

**Table:**

C_24_H_35_N_2_O_5_^+^·Cl^−^·2H_2_O	*F*(000) = 1080
*M**_r_* = 503.02	*D*_x_ = 1.218 Mg m^−^^3^
Monoclinic, *P*2_1_/*c*	Melting point: 406(2) K
Hall symbol: -P 2ybc	Cu *K*α radiation, λ = 1.54184 Å
*a* = 21.977 (3) Å	Cell parameters from 3039 reflections
*b* = 12.2295 (10) Å	θ = 3.6–67.2°
*c* = 10.2217 (9) Å	µ = 1.59 mm^−^^1^
β = 93.490 (9)°	*T* = 295 K
*V* = 2742.2 (5) Å^3^	Prism, colourless
*Z* = 4	0.60 × 0.40 × 0.35 mm

### Data collection

**Table:**

Oxford Diffraction Xcalibur Ruby diffractometer	4860 independent reflections
Radiation source: Enhance (Cu) X-ray Source	3012 reflections with *I* > 2σ(*I*)
Graphite monochromator	*R*_int_ = 0.035
Detector resolution: 10.2576 pixels mm^-1^	θ_max_ = 67.3°, θ_min_ = 4.0°
ω scans	*h* = −25→26
Absorption correction: multi-scan (*CrysAlis PRO*; Oxford Diffraction, 2009)	*k* = −14→12
*T*_min_ = 0.599, *T*_max_ = 1.000	*l* = −12→12
11225 measured reflections	

### Refinement

**Table:**

Refinement on *F*^2^	Primary atom site location: structure-invariant direct methods
Least-squares matrix: full	Secondary atom site location: difference Fourier map
*R*[*F*^2^ > 2σ(*F*^2^)] = 0.046	Hydrogen site location: inferred from neighbouring sites
*wR*(*F*^2^) = 0.131	H atoms treated by a mixture of independent and constrained refinement
*S* = 0.97	*w* = 1/[σ^2^(*F*_o_^2^) + (0.0748*P*)^2^] where *P* = (*F*_o_^2^ + 2*F*_c_^2^)/3
4860 reflections	(Δ/σ)_max_ = 0.001
339 parameters	Δρ_max_ = 0.20 e Å^−^^3^
0 restraints	Δρ_min_ = −0.24 e Å^−^^3^

### Special details

**Table:**

Geometry. All e.s.d.'s (except the e.s.d. in the dihedral angle between two l.s. planes) are estimated using the full covariance matrix. The cell e.s.d.'s are taken into account individually in the estimation of e.s.d.'s in distances, angles and torsion angles; correlations between e.s.d.'s in cell parameters are only used when they are defined by crystal symmetry. An approximate (isotropic) treatment of cell e.s.d.'s is used for estimating e.s.d.'s involving l.s. planes.
Refinement. Refinement of *F*^2^ against ALL reflections. The weighted *R*-factor *wR* and goodness of fit *S* are based on *F*^2^, conventional *R*-factors *R* are based on *F*, with *F* set to zero for negative *F*^2^. The threshold expression of *F*^2^ > σ(*F*^2^) is used only for calculating *R*-factors(gt) *etc*. and is not relevant to the choice of reflections for refinement. *R*-factors based on *F*^2^ are statistically about twice as large as those based on *F*, and *R*- factors based on ALL data will be even larger.

### Fractional atomic coordinates and isotropic or equivalent isotropic displacement parameters (Å^2^)

**Table:**

	*x*	*y*	*z*	*U*_iso_*/*U*_eq_	
Cl1	0.60316 (3)	0.54002 (5)	0.43730 (7)	0.0681 (2)	
O1	0.04553 (8)	0.60760 (16)	−0.00797 (18)	0.0712 (5)	
O2	0.04433 (7)	0.62103 (15)	0.24413 (18)	0.0680 (5)	
O3	0.35306 (8)	0.87255 (13)	0.4546 (2)	0.0689 (5)	
O4	0.83315 (9)	1.01542 (13)	0.90785 (19)	0.0709 (5)	
O5	0.89012 (7)	0.84794 (15)	1.01019 (17)	0.0636 (5)	
N1	0.33989 (9)	0.69124 (17)	0.4423 (2)	0.0553 (5)	
N2	0.59396 (8)	0.76971 (17)	0.5691 (2)	0.0460 (5)	
C1	0.21020 (10)	0.58872 (18)	0.2146 (2)	0.0503 (6)	
C2	0.21035 (11)	0.5805 (2)	0.0803 (2)	0.0584 (6)	
H2A	0.2469	0.5712	0.0405	0.070*	
C3	0.15577 (12)	0.5863 (2)	0.0040 (2)	0.0605 (6)	
H3A	0.1565	0.5803	−0.0866	0.073*	
C4	0.10113 (11)	0.60055 (19)	0.0590 (2)	0.0520 (6)	
C5	0.10050 (10)	0.60831 (18)	0.1960 (2)	0.0498 (6)	
C6	0.15446 (10)	0.60152 (19)	0.2707 (2)	0.0504 (6)	
H6A	0.1538	0.6056	0.3614	0.061*	
C7	0.04354 (15)	0.5862 (3)	−0.1458 (3)	0.0874 (10)	
H7A	0.0020	0.5880	−0.1807	0.131*	
H7B	0.0606	0.5153	−0.1608	0.131*	
H7C	0.0667	0.6408	−0.1883	0.131*	
C8	0.03998 (14)	0.6059 (3)	0.3813 (3)	0.0794 (9)	
H8A	−0.0021	0.6062	0.4014	0.119*	
H8B	0.0611	0.6641	0.4279	0.119*	
H8C	0.0581	0.5372	0.4072	0.119*	
C9	0.26858 (11)	0.58427 (19)	0.3007 (3)	0.0574 (6)	
H9A	0.3003	0.5506	0.2525	0.069*	
H9B	0.2623	0.5393	0.3768	0.069*	
C10	0.28909 (10)	0.69740 (19)	0.3453 (3)	0.0557 (6)	
H10A	0.3011	0.7391	0.2704	0.067*	
H10B	0.2554	0.7352	0.3824	0.067*	
C11	0.36817 (10)	0.77996 (19)	0.4917 (2)	0.0503 (6)	
C12	0.42055 (10)	0.76099 (19)	0.5905 (3)	0.0514 (6)	
H12A	0.4174	0.6882	0.6273	0.062*	
H12B	0.4185	0.8134	0.6613	0.062*	
C13	0.48157 (10)	0.7724 (2)	0.5283 (2)	0.0544 (6)	
H13A	0.4860	0.7135	0.4661	0.065*	
H13B	0.4824	0.8410	0.4808	0.065*	
C14	0.53415 (10)	0.7694 (2)	0.6305 (2)	0.0519 (6)	
H14A	0.5319	0.8324	0.6876	0.062*	
H14B	0.5311	0.7041	0.6836	0.062*	
C15	0.64681 (10)	0.7750 (2)	0.6672 (2)	0.0548 (6)	
H15A	0.6467	0.7105	0.7225	0.066*	
H15B	0.6425	0.8385	0.7226	0.066*	
C16	0.70733 (10)	0.7816 (2)	0.6030 (3)	0.0602 (7)	
H16A	0.7139	0.7152	0.5539	0.072*	
H16B	0.7068	0.8428	0.5426	0.072*	
C17	0.75800 (10)	0.7962 (2)	0.7074 (2)	0.0518 (6)	
C18	0.77208 (10)	0.9006 (2)	0.7562 (2)	0.0516 (6)	
H18A	0.7513	0.9609	0.7205	0.062*	
C19	0.81614 (10)	0.91564 (18)	0.8559 (2)	0.0502 (6)	
C20	0.84737 (10)	0.82529 (19)	0.9117 (2)	0.0491 (6)	
C21	0.83303 (11)	0.72290 (19)	0.8639 (3)	0.0604 (7)	
H21A	0.8532	0.6621	0.8999	0.072*	
C22	0.78904 (11)	0.7091 (2)	0.7631 (3)	0.0621 (7)	
H22A	0.7803	0.6390	0.7321	0.074*	
C23	0.79880 (15)	1.1081 (2)	0.8642 (3)	0.0781 (9)	
H23A	0.8149	1.1723	0.9079	0.117*	
H23B	0.7570	1.0984	0.8838	0.117*	
H23C	0.8013	1.1163	0.7713	0.117*	
C24	0.91979 (13)	0.7568 (2)	1.0737 (3)	0.0776 (9)	
H24A	0.9487	0.7826	1.1408	0.116*	
H24B	0.9406	0.7150	1.0106	0.116*	
H24C	0.8900	0.7116	1.1123	0.116*	
O1W	0.46801 (16)	0.5117 (2)	0.3085 (3)	0.0915 (7)	
O2W	0.40073 (12)	1.04928 (17)	0.5826 (3)	0.0892 (8)	
H1W2	0.3843 (19)	0.987 (4)	0.537 (4)	0.159 (17)*	
H2W2	0.4236 (18)	1.034 (3)	0.644 (4)	0.116 (15)*	
H1W1	0.448 (2)	0.495 (4)	0.376 (5)	0.16 (2)*	
H2W1	0.509 (2)	0.513 (4)	0.330 (4)	0.16 (2)*	
H1	0.3518 (11)	0.629 (2)	0.476 (2)	0.058 (7)*	
H2	0.5971 (11)	0.707 (2)	0.520 (3)	0.071 (8)*	
H3	0.5953 (10)	0.834 (2)	0.511 (2)	0.062 (7)*	

### Atomic displacement parameters (Å^2^)

**Table:**

	*U*^11^	*U*^22^	*U*^33^	*U*^12^	*U*^13^	*U*^23^
Cl1	0.0868 (5)	0.0462 (3)	0.0703 (4)	0.0017 (3)	−0.0030 (3)	0.0004 (3)
O1	0.0576 (11)	0.0906 (14)	0.0628 (11)	−0.0020 (10)	−0.0185 (9)	0.0016 (10)
O2	0.0452 (10)	0.0919 (14)	0.0668 (12)	0.0045 (9)	0.0015 (8)	−0.0032 (10)
O3	0.0586 (11)	0.0429 (10)	0.1020 (15)	0.0001 (8)	−0.0209 (10)	0.0039 (9)
O4	0.0856 (13)	0.0493 (10)	0.0745 (12)	0.0019 (9)	−0.0230 (10)	−0.0066 (9)
O5	0.0515 (10)	0.0699 (11)	0.0673 (11)	−0.0008 (8)	−0.0153 (8)	0.0052 (9)
N1	0.0438 (11)	0.0415 (11)	0.0781 (15)	−0.0004 (9)	−0.0155 (10)	0.0037 (11)
N2	0.0391 (11)	0.0452 (11)	0.0529 (12)	−0.0006 (8)	−0.0044 (9)	−0.0062 (10)
C1	0.0465 (13)	0.0435 (12)	0.0599 (15)	0.0000 (10)	−0.0047 (11)	−0.0039 (11)
C2	0.0507 (14)	0.0662 (16)	0.0585 (16)	−0.0026 (12)	0.0052 (12)	−0.0041 (13)
C3	0.0652 (17)	0.0707 (16)	0.0451 (14)	−0.0081 (14)	−0.0002 (12)	−0.0010 (12)
C4	0.0505 (14)	0.0507 (14)	0.0535 (15)	−0.0061 (11)	−0.0074 (11)	0.0030 (11)
C5	0.0455 (13)	0.0496 (13)	0.0537 (14)	0.0012 (10)	−0.0018 (11)	−0.0045 (11)
C6	0.0498 (13)	0.0551 (14)	0.0456 (13)	−0.0004 (11)	−0.0031 (11)	−0.0037 (11)
C7	0.092 (2)	0.104 (2)	0.0616 (19)	−0.0084 (19)	−0.0325 (17)	−0.0010 (17)
C8	0.0722 (19)	0.095 (2)	0.072 (2)	0.0049 (16)	0.0203 (15)	0.0011 (17)
C9	0.0493 (14)	0.0501 (13)	0.0713 (17)	0.0025 (11)	−0.0085 (12)	−0.0103 (13)
C10	0.0440 (13)	0.0493 (14)	0.0720 (17)	−0.0024 (11)	−0.0109 (12)	0.0022 (12)
C11	0.0389 (12)	0.0466 (14)	0.0648 (16)	−0.0013 (10)	−0.0008 (11)	0.0000 (11)
C12	0.0421 (13)	0.0456 (13)	0.0652 (16)	−0.0010 (10)	−0.0056 (11)	−0.0037 (11)
C13	0.0417 (13)	0.0599 (15)	0.0606 (15)	0.0026 (11)	−0.0051 (11)	−0.0062 (12)
C14	0.0386 (12)	0.0548 (14)	0.0615 (15)	0.0028 (10)	−0.0023 (11)	−0.0094 (11)
C15	0.0411 (13)	0.0677 (16)	0.0540 (14)	−0.0034 (11)	−0.0093 (11)	−0.0035 (12)
C16	0.0453 (14)	0.0749 (18)	0.0596 (15)	−0.0038 (12)	−0.0036 (12)	−0.0055 (13)
C17	0.0370 (12)	0.0616 (15)	0.0564 (15)	−0.0028 (11)	−0.0010 (11)	−0.0014 (12)
C18	0.0451 (13)	0.0548 (14)	0.0541 (14)	0.0052 (11)	−0.0032 (11)	0.0044 (11)
C19	0.0481 (13)	0.0471 (13)	0.0549 (14)	−0.0011 (11)	0.0006 (11)	0.0005 (11)
C20	0.0364 (12)	0.0562 (14)	0.0544 (14)	−0.0015 (10)	−0.0005 (11)	0.0044 (11)
C21	0.0458 (14)	0.0509 (15)	0.0831 (19)	0.0057 (11)	−0.0064 (13)	0.0089 (13)
C22	0.0499 (14)	0.0490 (14)	0.086 (2)	−0.0052 (12)	−0.0043 (14)	−0.0047 (13)
C23	0.122 (3)	0.0499 (16)	0.0619 (18)	0.0174 (16)	0.0018 (17)	−0.0008 (13)
C24	0.0581 (17)	0.097 (2)	0.076 (2)	0.0129 (15)	−0.0156 (15)	0.0192 (17)
O1W	0.104 (2)	0.0987 (17)	0.0698 (15)	−0.0214 (15)	−0.0146 (14)	0.0143 (12)
O2W	0.1143 (19)	0.0535 (12)	0.0948 (18)	−0.0154 (12)	−0.0341 (15)	0.0146 (12)

### Geometric parameters (Å, º)

**Table:**

O1—C4	1.366 (3)	C10—H10B	0.9700
O1—C7	1.431 (3)	C11—C12	1.503 (3)
O2—C5	1.365 (3)	C12—C13	1.525 (3)
O2—C8	1.423 (3)	C12—H12A	0.9700
O3—C11	1.233 (3)	C12—H12B	0.9700
O4—C19	1.374 (3)	C13—C14	1.510 (3)
O4—C23	1.419 (3)	C13—H13A	0.9700
O5—C20	1.363 (3)	C13—H13B	0.9700
O5—C24	1.427 (3)	C14—H14A	0.9700
N1—C11	1.334 (3)	C14—H14B	0.9700
N1—C10	1.449 (3)	C15—C16	1.521 (3)
N1—H1	0.86 (2)	C15—H15A	0.9700
N2—C15	1.488 (3)	C15—H15B	0.9700
N2—C14	1.490 (3)	C16—C17	1.505 (3)
N2—H2	0.92 (3)	C16—H16A	0.9700
N2—H3	0.99 (3)	C16—H16B	0.9700
C1—C2	1.377 (3)	C17—C22	1.369 (3)
C1—C6	1.392 (3)	C17—C18	1.398 (3)
C1—C9	1.512 (3)	C18—C19	1.375 (3)
C2—C3	1.392 (3)	C18—H18A	0.9300
C2—H2A	0.9300	C19—C20	1.404 (3)
C3—C4	1.368 (3)	C20—C21	1.374 (3)
C3—H3A	0.9300	C21—C22	1.380 (3)
C4—C5	1.405 (3)	C21—H21A	0.9300
C5—C6	1.373 (3)	C22—H22A	0.9300
C6—H6A	0.9300	C23—H23A	0.9600
C7—H7A	0.9600	C23—H23B	0.9600
C7—H7B	0.9600	C23—H23C	0.9600
C7—H7C	0.9600	C24—H24A	0.9600
C8—H8A	0.9600	C24—H24B	0.9600
C8—H8B	0.9600	C24—H24C	0.9600
C8—H8C	0.9600	O1W—H1W1	0.86 (5)
C9—C10	1.517 (3)	O1W—H2W1	0.91 (5)
C9—H9A	0.9700	O2W—H1W2	0.96 (4)
C9—H9B	0.9700	O2W—H2W2	0.80 (4)
C10—H10A	0.9700		
			
C4—O1—C7	117.0 (2)	C13—C12—H12A	109.4
C5—O2—C8	117.19 (19)	C11—C12—H12B	109.4
C19—O4—C23	117.45 (19)	C13—C12—H12B	109.4
C20—O5—C24	116.9 (2)	H12A—C12—H12B	108.0
C11—N1—C10	122.6 (2)	C14—C13—C12	111.4 (2)
C11—N1—H1	116.1 (16)	C14—C13—H13A	109.3
C10—N1—H1	121.2 (16)	C12—C13—H13A	109.3
C15—N2—C14	112.89 (19)	C14—C13—H13B	109.3
C15—N2—H2	108.8 (16)	C12—C13—H13B	109.3
C14—N2—H2	108.9 (16)	H13A—C13—H13B	108.0
C15—N2—H3	108.8 (13)	N2—C14—C13	111.5 (2)
C14—N2—H3	108.5 (14)	N2—C14—H14A	109.3
H2—N2—H3	109 (2)	C13—C14—H14A	109.3
C2—C1—C6	118.3 (2)	N2—C14—H14B	109.3
C2—C1—C9	121.6 (2)	C13—C14—H14B	109.3
C6—C1—C9	120.1 (2)	H14A—C14—H14B	108.0
C1—C2—C3	120.0 (2)	N2—C15—C16	112.3 (2)
C1—C2—H2A	120.0	N2—C15—H15A	109.2
C3—C2—H2A	120.0	C16—C15—H15A	109.2
C4—C3—C2	121.6 (2)	N2—C15—H15B	109.2
C4—C3—H3A	119.2	C16—C15—H15B	109.2
C2—C3—H3A	119.2	H15A—C15—H15B	107.9
O1—C4—C3	125.7 (2)	C17—C16—C15	109.2 (2)
O1—C4—C5	115.6 (2)	C17—C16—H16A	109.8
C3—C4—C5	118.7 (2)	C15—C16—H16A	109.8
O2—C5—C6	125.1 (2)	C17—C16—H16B	109.8
O2—C5—C4	115.5 (2)	C15—C16—H16B	109.8
C6—C5—C4	119.3 (2)	H16A—C16—H16B	108.3
C5—C6—C1	122.0 (2)	C22—C17—C18	117.9 (2)
C5—C6—H6A	119.0	C22—C17—C16	122.1 (2)
C1—C6—H6A	119.0	C18—C17—C16	119.9 (2)
O1—C7—H7A	109.5	C19—C18—C17	121.1 (2)
O1—C7—H7B	109.5	C19—C18—H18A	119.4
H7A—C7—H7B	109.5	C17—C18—H18A	119.4
O1—C7—H7C	109.5	O4—C19—C18	124.6 (2)
H7A—C7—H7C	109.5	O4—C19—C20	115.3 (2)
H7B—C7—H7C	109.5	C18—C19—C20	120.1 (2)
O2—C8—H8A	109.5	O5—C20—C21	125.5 (2)
O2—C8—H8B	109.5	O5—C20—C19	116.0 (2)
H8A—C8—H8B	109.5	C21—C20—C19	118.5 (2)
O2—C8—H8C	109.5	C20—C21—C22	120.8 (2)
H8A—C8—H8C	109.5	C20—C21—H21A	119.6
H8B—C8—H8C	109.5	C22—C21—H21A	119.6
C1—C9—C10	111.61 (19)	C17—C22—C21	121.6 (2)
C1—C9—H9A	109.3	C17—C22—H22A	119.2
C10—C9—H9A	109.3	C21—C22—H22A	119.2
C1—C9—H9B	109.3	O4—C23—H23A	109.5
C10—C9—H9B	109.3	O4—C23—H23B	109.5
H9A—C9—H9B	108.0	H23A—C23—H23B	109.5
N1—C10—C9	111.15 (19)	O4—C23—H23C	109.5
N1—C10—H10A	109.4	H23A—C23—H23C	109.5
C9—C10—H10A	109.4	H23B—C23—H23C	109.5
N1—C10—H10B	109.4	O5—C24—H24A	109.5
C9—C10—H10B	109.4	O5—C24—H24B	109.5
H10A—C10—H10B	108.0	H24A—C24—H24B	109.5
O3—C11—N1	121.3 (2)	O5—C24—H24C	109.5
O3—C11—C12	122.0 (2)	H24A—C24—H24C	109.5
N1—C11—C12	116.7 (2)	H24B—C24—H24C	109.5
C11—C12—C13	111.3 (2)	H1W1—O1W—H2W1	111 (4)
C11—C12—H12A	109.4	H1W2—O2W—H2W2	113 (3)

### Hydrogen-bond geometry (Å, º)

**Table:**

*D*—H···*A*	*D*—H	H···*A*	*D*···*A*	*D*—H···*A*
N1—H1···Cl1^i^	0.87 (2)	2.44 (2)	3.300 (2)	173 (2)
O2*W*—H1*W*2···O3	0.95 (5)	1.75 (5)	2.704 (3)	179 (6)
N2—H2···Cl1	0.92 (3)	2.22 (3)	3.127 (2)	169 (3)
O2*W*—H2*W*2···O1*W*^ii^	0.80 (4)	1.97 (4)	2.767 (4)	171 (4)
N2—H3···O2*W*^iii^	0.99 (2)	1.72 (2)	2.709 (3)	177 (2)
O1*W*—H1*W*1···Cl1^i^	0.87 (5)	2.31 (5)	3.177 (3)	177 (6)
O1*W*—H2*W*1···Cl1	0.91 (4)	2.31 (4)	3.194 (4)	164 (3)

Symmetry codes: (i) −*x*+1, −*y*+1, −*z*+1; (ii) *x*, −*y*+3/2, *z*+1/2; (iii) −*x*+1, −*y*+2, −*z*+1.
